# Real-World Experience With Panitumumab or Bevacizumab as a First-Line Treatment for Metastatic Colorectal Cancer: An Indian Perspective

**DOI:** 10.7759/cureus.97914

**Published:** 2025-11-27

**Authors:** Ghanshyam Biswas, Sumit Goyal, Amit Rauthan, Syed Mujtaba Hussain Naqvi, Sunil Kumar Yadav Yadagiri, Gauri Debashish Dhanaki, Arti Sanghavi, Rohit Kumar, Sagar Katare, Bhavesh P Kotak

**Affiliations:** 1 Medical Oncology, Sparsh Hospitals &amp; Critical Care, Bhubaneswar, IND; 2 Medical Oncology, Rajiv Gandhi Cancer Institute and Research Centre, New Delhi, IND; 3 Medical Oncology, Manipal Hospital, Bengaluru, IND; 4 Medical Affairs, Dr. Reddy's Laboratories, Hyderabad, IND; 5 Clinical Research, Dr. Reddy's Laboratories, Hyderabad, IND

**Keywords:** bevacizumab, colorectal cancer, metastatic, panitumumab, real-world evidence

## Abstract

Objective

This study aimed to evaluate the effectiveness and safety of panitumumab and bevacizumab in conjunction with first-line chemotherapy in Indian patients with metastatic colorectal cancer (mCRC).

Materials and methods

A retrospective chart review was performed involving consecutive patients treated between September 2015 and December 2023. Study endpoints included comparisons of the objective response rate (ORR), clinical benefit rate (CBR), progression-free survival (PFS), overall survival (OS), and adverse drug reactions associated with bevacizumab- and panitumumab-based regimens, along with assessments of the clinical profiles and tumor characteristics of patients with mCRC.

Results

This study analyzed data from 69 patients, including 30 in the panitumumab group and 39 in the bevacizumab group. The cohort consisted of 42 males (60.9%) and 27 females (39.1%), with a mean age of 54.0 ± 14.05 years. KRAS mutations were identified in 10.2% of patients. No significant differences were observed between the panitumumab and bevacizumab groups in ORR (70% vs. 69.23%, p=0.94), CBR (83.33% vs. 87.18%, p=0.65), or PFS (8.43 vs. 8.17 months, p=0.15). Median OS was 26.13 months in the bevacizumab group, while it was not reached in the panitumumab group at a median follow-up of 15.02 months. Adverse events were more frequent in the bevacizumab group. Additionally, no significant differences in clinical outcomes were found between bevacizumab- and panitumumab-based regimens for tumors originating on either the left or right side.

Conclusions

The results of the study show that bevacizumab and panitumumab-based regimens provide comparable outcomes in Indian patients with mCRC.

## Introduction

Colorectal cancer (CRC) is a prevalent gastrointestinal condition, ranking third in the number of newly diagnosed cases and second in the number of tumor-related deaths worldwide [1]. The prevalence of colon cancer in India continues to rise, with rates ranging from 20% to 124% per year, according to cancer registries [2]. Studies show that 20% of CRC patients present with metastatic disease at diagnosis, and more than half of those initially diagnosed with localized cancer eventually develop metastases during treatment, resulting in a five-year survival rate below 5% [3-5]. Additionally, after surgery, 50-60% of patients may have a recurrence that might advance to an unresectable stage [6]. According to studies, patients with early- and middle-stage disease have an overall survival (OS) rate of more than 60% at five years following surgery, compared to less than a year for patients with advanced CRC [7]. In India, the five-year survival rate for CRC is the lowest in the world, with a rate of less than 40% [8].

Surgery and adjuvant chemotherapy are the primary treatments for advanced CRC. First-line therapy typically includes multi-agent regimens like FOLFOX, FOLFIRI, or CAPOX, which offer superior progression-free survival (PFS) and OS compared to fluoropyrimidine monotherapy [9-12]. Furthermore, studies have demonstrated the potential use of targeted therapies, such as vascular endothelial growth factor (VEGF) and epidermal growth factor receptor (EGFR) inhibitors, in conjunction with conventional chemotherapy since metastatic CRC (mCRC) exhibits several genetic alterations [7]. Bevacizumab, initially approved for mCRC in 2004, was later incorporated into second-line therapy due to its demonstrated survival benefit [3]. When combined with chemotherapy as first-line treatment, it improves survival outcomes in mCRC [13-15]. RAS and BRAF mutations are key prognostic and predictive markers. EGFR inhibitors, such as cetuximab and panitumumab, are effective in KRAS/NRAS wild-type tumors, and panitumumab plus best supportive care (BSC) has been shown to improve PFS in chemotherapy-refractory mCRC [16]. Moreover, patients with left-sided RAS wild-type tumors exhibit significantly better OS compared with those with right-sided tumors [17].

A meta-analysis reported that in KRAS/RAS wild-type (WT) mCRC, EGFR inhibitors significantly improved overall survival (OS, p=0.003) and response rates (p=0.0003) compared with VEGF inhibitors, without a significant effect on PFS [18]. Another meta-analysis compared first-line EGFR inhibitors plus FOLFIRI/FOLFOX with bevacizumab plus chemotherapy in RAS WT mCRC patients [19]. For left-sided tumors, objective response rate (ORR) and OS favored EGFR inhibitors plus chemotherapy, while for right-sided tumors, PFS favored bevacizumab plus chemotherapy. Additionally, panitumumab showed improved OS over bevacizumab in left-sided and overall RAS WT mCRC when used with standard first-line therapy [20]. Despite these findings, direct comparisons of first-line bevacizumab and panitumumab in mCRC are limited, particularly in the Indian population. This retrospective study, therefore, aimed to evaluate the efficacy and safety of these agents in combination with first-line chemotherapy in Indian patients with mCRC.

## Materials and methods

This multi-centric, observational, real-world evidence study was conducted at three centers in India. A retrospective chart review was undertaken for consecutive patients who received panitumumab or bevacizumab as first-line treatment for their mCRC between September 2015 and December 2023. The eligibility criteria included male and female patients over the age of 18 years, a histologically confirmed diagnosis of mCRC, and first-line therapy with panitumumab or bevacizumab targeted therapies. Patients were excluded if they had less than six months of follow-up or if their targeted therapy had commenced prior to 2015. The selection of bevacizumab versus panitumumab reflected routine clinical practice and was determined at the discretion of the treating oncologist.

Selection was guided by commonly considered real-world factors, including RAS status, tumor sidedness, comorbidities, anticipated toxicity profiles, patient affordability, and overall clinical judgment. Both biologic agents were administered as per institutional standard dosing protocols, and any dose modifications or treatment duration adjustments were carried out according to routine clinical practice. The study was carried out under the applicable guidelines for good clinical practice as well as the ethical principles outlined in the most recent version of the Helsinki Declaration. Every participating center obtained approval from its institutional ethics committee, and the study was registered on the Clinical Trials Registry of India portal under registration number CTRI/2023/09/057410.

Data collection, response assessment, and statistical analysis

Deidentified data for the patient characteristics (age, gender, clinical details, comorbid conditions, and performance status), tumor characteristics (initial diagnosis, stage, clinical presentation, colonoscopy findings, site of the tumor, histology, morphology, molecular profiling, and sites of metastasis), treatment details (chemotherapy, targeted therapy, dose, number of cycles, response, and toxicity), and survival status was collected from the medical records.

The clinical and radiological assessments served as the basis for evaluating the response. ORR was defined as the proportion of patients achieving a complete response (CR) or partial response (PR) among all patients evaluable for response. The clinical benefit rate (CBR) included patients with CR, PR, or stable disease (SD) as their best response, relative to the total number of evaluable patients. PFS was defined as the time from treatment initiation to the first documented progressive disease (PD) or death from any cause. For patients without PD at the time of data collection, PFS was censored at the date of the last progression-free assessment. If a patient received another anticancer therapy before PD or death, PFS was censored at the date of the last progression-free observation before the new treatment. OS was measured from the start of treatment to death from any cause, with surviving patients censored at the date of their last follow-up.

All data were recorded on a predesigned proforma. The categorical variables were summarized using frequencies and percentages. The Chi-square or Fisher's exact test was utilized to examine the proportion between the groups. Approximate normalcy was assessed for quantitative variables. To describe variables with a normal distribution, the mean and standard deviation were employed. Non-normal variables were summarized as median (minimum, maximum), and Friedman's test, followed by the Wilcoxon rank sum test for pairwise comparison, if required, was used to compare the distribution between groups. Kaplan-Meier survival analysis was used to draw the time to the probability of time to event curve (PFS and OS) for each group. The log-rank test was used to compare the time-to-event curve between the groups. All statistical tests were two-sided. By compiling the frequency of adverse events in the study population, an assessment of safety was carried out. The data were analyzed using Stata 16.0 statistical software (StataCorp LLC, College Station, TX).

## Results

The study initially collected data from 80 patients. However, 11 were excluded due to off-label use of targeted therapies. Consequently, 69 patients remained eligible for analysis, including 30 in the panitumumab group and 39 in the bevacizumab group.

Descriptive characteristics

There were 42 males (60.9%) and 27 females (39.1%), with a mean age of 54.0 ± 14.05 years. More than 95% of patients had an Eastern Cooperative Oncology Group (ECOG) performance status of ≤2. Among the 69 participants, 47 (68.1%) had left-sided tumors, 22 (31.9%) had right-sided tumors, and 47 (68.1%) presented with de novo mCRC. Metastatic sites included the liver (66.7%), lungs (40.6%), lymph nodes (31.9%), bones (15.9%), and peritoneum (17.4%). Table 1 summarizes the descriptive characteristics of the study population.

**Table 1:** Descriptive characteristics of the study population (N=69) SD: standard deviation; BMI: body mass index; CEA: carcinoembryonic antigen; ECOG: Eastern Cooperative Oncology Group; CRC: colorectal cancer

Variable	Values
Age, years, mean ± SD	54.00 ± 14.05
Sex, n (%)	
Female	27 (39.1)
Male	42 (60.9)
BMI, kg/m^2^, mean ± SD	24.33 ± 4.83
CEA, ng/ml, median (range)	19.10 (0.59 - 3300)
ECOG ≤2, n (%)	66 (95.7)
Comorbidities, n (%)	
Hypertension	22 (31.9)
Diabetes	14 (20.3)
Hypothyroid	3 (4.3)
Other	11 (15.9)
Colonoscopy findings	
Ulcerated	21 (30.4)
Circumferential	19 (27.5)
Polyp	15 (21.7)
Cauliflower	1 (1.4)
Not available	13 (18.8)
Site of tumor, n (%)	
Caecum	2 (2.9)
Ascending colon	8 (11.6)
Hepatic flexure	6 (8.7)
Transverse colon	6 (8.7)
Splenic flexure	1 (1.4)
Descending colon	3 (4.3)
Sigmoid colon	26 (37.7)
Rectosigmoid junction	6 (8.7)
Rectum	11 (15.9)
Location of tumor, n (%)	
Right	22 (31.9)
Left	47 (68.1)
Histology, n (%)	
Mucinous	12 (17.4)
Signet ring type	9 (13.0)
Tubular adenoma	1 (1.4)
Tubulovillous	1 (1.4)
Intramucosal	1 (1.4)
Unknown	45 (65.2)
Morphology, n (%)	
Well differentiated	5 (7.2)
Moderately differentiated	33 (47.8)
Poorly differentiated	18 (26.1)
Unknown	13 (18.8)
Diagnosis, n (%)	
Previously treated localized CRC and now diagnosed as metastatic disease	22 (31.9)
De novo metastatic CRC	47 (68.1)
Sites of metastasis, n (%)	
Liver	46 (66.7)
Lung	28 (40.6)
Lymph nodes	22 (31.9)
Bone	11 (15.9)
Peritoneum	12 (17.4)
Omentum	4 (5.8)
Ovary	4 (5.8)
Others	7 (10.1)
First treatment, n (%)	
Previously treated	
Surgical resection	21 (30.4)
Radiotherapy	1 (1.4)
Denovo	47 (68.1)
Status at last follow-up, n (%)	
Alive	54 (78.3)
Dead	15 (21.7)

Treatment distribution based on molecular profile and tumor sidedness

Table 2 presents the treatments administered according to molecular profile and tumor sidedness.

**Table 2:** Treatment distribution based on molecular profile and tumor sidedness KRAS: Kirsten rat sarcoma viral oncogene; NRAS: neuroblastoma-RAS; BRAF: V-Raf murine sarcoma viral oncogene homolog B1; HER2: human epidermal growth factor receptor 2; MSI: microsatellite instability; MSS: microsatellite stable; MMR: mismatch repair

Molecular profile (N)	Mutational status, n (%)	Left-sided tumors			Right-sided tumors		
		N (%)	Panitumumab	Bevacizumab	N (%)	Panitumumab	Bevacizumab
KRAS (59)	Wild type: 53 (89.8)	36 (67.9)	27 (75.0)	9 (25.0)	17 (32.1)	2 (11.8)	15 (88.2)
	Mutant: 6 (10.2)	5 (83.3)	-	5 (100.0)	1 (16.7)	-	1 (100.0)
NRAS (58)	Wild type: 58 (100.0)	41 (70.7)	27 (65.9)	14 (34.1)	17 (29.3)	2 (11.8)	15 (88.2)
	Mutant: Nil	-	-	-	-	-	-
BRAF (41)	Wild type: 41 (100.0)	30 (73.2)	22 (73.3)	8 (26.7)	11 (26.8)	1 (9.1)	10 (90.9)
	Mutant: Nil	-	-	-	-	-	-
HER2 (40)	Positive: 2 (5.0)	2 (100.0)	1 (50.0)	1 (50.0)	-	-	-
	Negative: 38 (95.0)	19 (50.0)	9 (47.4)	10 (52.6)	19 (50.0)	2 (10.5)	17 (89.5)
MSI (27)	High: 6 (22.2)	3 (50.0)	-	3 (100.0)	3 (50.0)	-	3 (100.0)
	Low: 9 (33.3)	5 (55.6)	2 (40.0)	3 (60.0)	4 (44.4)	-	4 (100.0)
	MSS: 12 (55.6)	3 (25.0)	-	3 (100.0)	9 (75.0)	-	9 (100.0)
MMR (25)	Proficient: 23 (92.0)	23 (100.0)	18 (78.3)	5 (21.7)	-	-	-
	Deficient: 2 (8.0)	1 (50.0)	1 (100.0)	-	1 (50.0)	-	1 (100.0)

The KRAS mutations were present in six (10.2%) patients. Among them, bevacizumab was administered to five patients (83.3%) with left-sided tumors and one patient (16.7%) with a right-sided tumor. Of the 53 patients (89.8%) with KRAS wild-type tumors, 36 (67.9%) had left-sided tumors, and 17 (32.1%) had right-sided tumors. Bevacizumab (88.2%) was the preferred therapy for right-sided tumors, whereas panitumumab (75.0%) was given to most left-sided KRAS WT tumors. HER2 was positive in two (5%) patients with left-sided tumors, one treated with panitumumab and the other with bevacizumab. Microsatellite instability (MSI) was high in six (22.2%) patients, three each in left and right-sided tumors, with all receiving bevacizumab therapy. The MMR gene was proficient in 23 (92%) patients with left-sided tumors: 18 (78.3%) and five (21.7%) of these were treated with panitumumab and bevacizumab, respectively.

Treatment details and response evaluation

Table 3 presents the treatment details and response assessment of panitumumab- and bevacizumab-based regimens.

**Table 3:** Treatment details and response assessment (N=69) Statistical test: Chi-square test and Fisher’s exact test PR: partial response; SD: stable disease; PD: progressive disease; ORR: objective response rate; CBR: clinical benefit rate; PFS: progression-free survival; OS: overall survival; CI: confidence interval

Characteristics	Panitumumab-based regimens	Bevacizumab-based regimens	Chi-square value	P-value
No. of patients	30	39	-	-
No. of cycles	8 (3-14)	8 (3-16)	-	-
Response, n (%)				
PR	21 (70.00)	27 (69.23)	-	-
SD	4 (13.33)	7 (17.95)	-	-
PD	5 (16.67)	5 (12.82)	-	-
ORR, n (%)	21 (70.00)	27 (69.23)	0.0047	0.94
CBR, n (%)	25 (83.33)	34 (87.18)	0.0202	0.65
PFS, months, median (95% CI)	8.43 (3.2-11.20)	8.17 (6.8-29.80)	2.09	0.1478
OS, months, median (95% CI)	At a median follow-up of 15.02 months, the median OS was not reached	26.13 (14.43-82.23)	-	-

**Figure 1:**
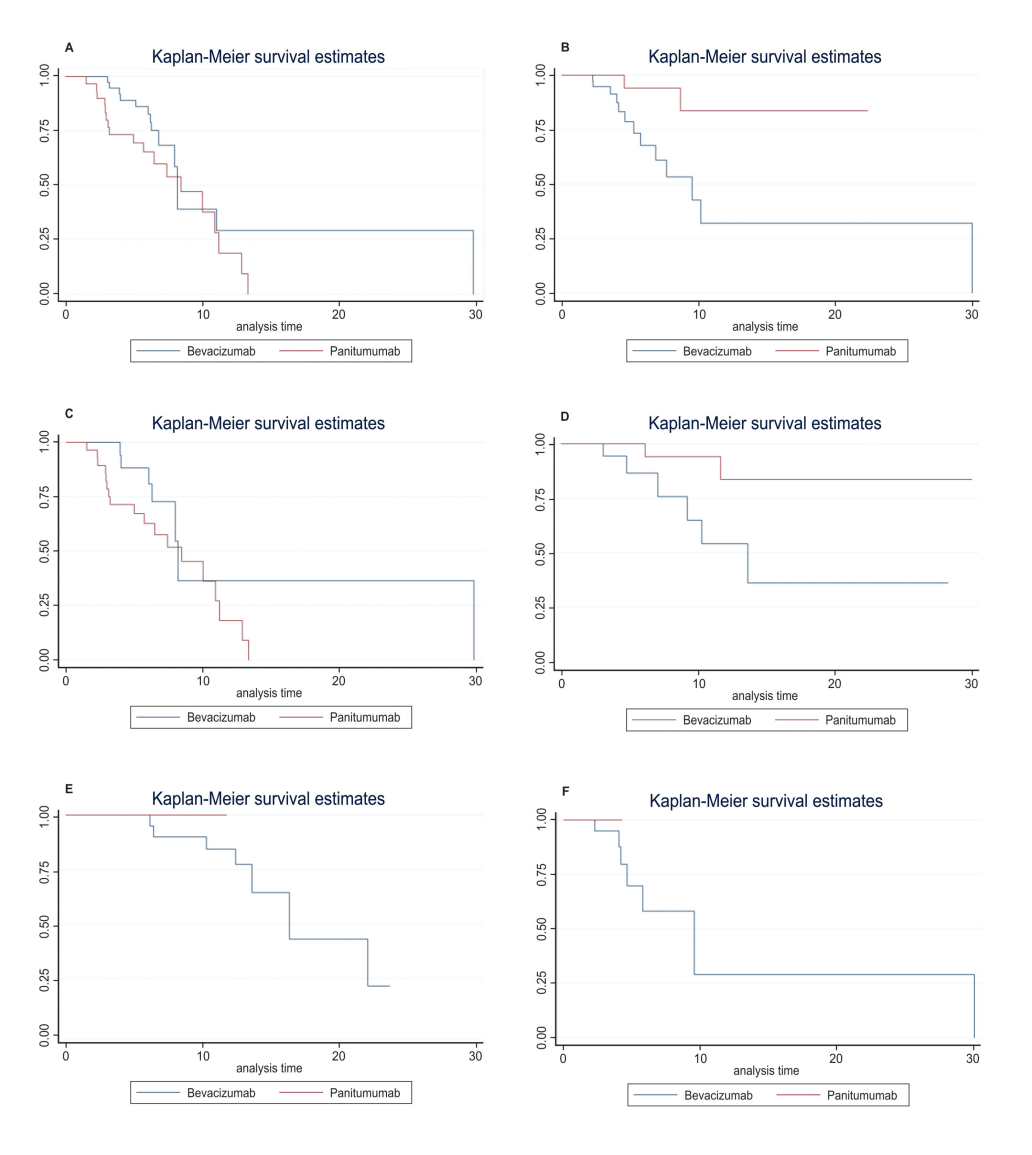
Kaplan-Meier survival curve for panitumumab versus bevacizumab (A) PFS for the overall population; (B) OS for the overall population; (C) PFS for left-sided tumors; (D) OS for left-sided tumors; (E) PFS for right-sided tumors; (F) OS for right-sided tumors PFS: progression-free survival; OS: overall survival

The ORR and CBR of first-line treatment with the panitumumab-based regimen were 70% and 83.33% respectively. The median number of treatment cycles administered was eight (range: 3-14), and the median PFS was 8.43 months (95% confidence interval (CI): 3.2-11.20 months; Figure 1A). The panitumumab-based regimen failed to achieve the median OS after 15.02 months of follow-up (Figure 1B). The ORR and CBR of the bevacizumab-based regimen were 69.23% and 87.18% respectively. The median number of treatment cycles was eight (range: 3-16). The median PFS and OS were 8.17 months (95% CI: 6.8-29.80 months; Figure 1A) and 26.13 months (95% CI: 14.43-82.23 months; Figure 1B). No significant differences were observed between the panitumumab and bevacizumab groups in terms of ORR, CBR, or PFS.

Treatment details and response assessment based on tumor sidedness

Table 4 presents a comparison of the response rates, PFS, and OS for panitumumab- and bevacizumab-based regimens for left- and right-sided tumors.

**Table 4:** Treatment details and response assessment of panitumumab- and bevacizumab-based regimens based on tumor sidedness Statistical test: Chi-square test and Fisher’s exact test ORR: objective response rate; CBR: clinical benefit rate; PFS: progression-free survival; OS: overall survival; CI: confidence interval; NE: not estimable

Characteristics	Panitumumab-based regimens	Bevacizumab-based regimens	Chi-square value	P-value
Left-sided tumors (n=47)				
No. of patients	28	19	-	-
No. of cycles	7.5 (3-14)	8 (4-16)	-	-
ORR, n (%)	19 (67.9)	15 (78.9)	0.695	0.40
CBR, n (%)	23 (82.1)	18 (94.7)	1.61	0.20
PFS, months, median (95% CI)	8.43 (3.1-11.20)	8.17 (6.27-29.8)	2.20	0.1383
OS, months, median (95% CI)	At a median follow-up of 15.90 months, the median OS was not reached	27.87 (18.83-NE)	-	-
Right-sided tumors (n=22)				
No. of patients	2	20	-	
No. of cycles	11 (10-12)	7 (3-12)	-	
ORR, n (%)	2 (100.0)	12 (60.0)	1.25	0.51
CBR, n (%)	2 (100.0)	16 (80.0)	0.488	1.0
PFS, months, median (95% CI)	At a median follow-up of 5.83 months, median PFS was not reached	8.17 (6.8-11.03)	-	-
OS, months, median (95% CI)	At a median follow-up of 11.25 months, the median OS was not reached	26.13 (12.67-82.23)	-	-

Out of 69 patients, 47 were diagnosed with left-sided tumors, with 28 of these patients receiving panitumumab and 19 receiving bevacizumab. There was no substantial difference in the ORR (67.9% vs. 78.9%, p=0.40) or CBR (82.1% vs. 94.7%, p=0.20) between the two groups for the left-sided tumors. Also, for the left-sided tumors, the median PFS did not differ significantly between the panitumumab group (8.43 months, 95% CI: 3.1-11.20) and the bevacizumab group (8.17 months, 95% CI: 6.27-29.80) (Figure 1C). For the left-sided tumors, the panitumumab group failed to achieve the median OS after 15.90 months of follow-up (Figure 1D). A total of 22 out of 69 patients had right-sided tumors, with two in the panitumumab group and 20 in the bevacizumab group. The two groups did not have a statistically significant difference in ORR (p=0.51) and CBR (p=1.0) for the right-sided tumors. The median PFS and OS, respectively, were not achieved by the panitumumab group at a median follow-up of 5.83 months and 11.25 months (Figures 1E, 1F). 

Adverse events

The adverse reactions associated with panitumumab- and bevacizumab-based regimens are listed in Table 5.

**Table 5:** Adverse events (N=69)

S. no.	Adverse events	Panitumumab-based regimens, n (%)	Bevacizumab-based regimens, n (%)
1.	Anorexia	3 (10.0)	4 (10.3)
2.	Generalized weakness	3 (10.0)	9 (23.1)
3.	Constipation	2 (6.7)	6 (15.4)
4.	Abdominal pain	1 (3.3)	2 (5.1)
5.	Anemia	1 (3.3)	1 (2.6)
6.	Fever	1 (3.3)	1 (2.6)
7.	Loose motions	1 (3.3)	1 (2.6)
8.	Vomiting	1 (3.3)	1 (2.6)
9.	Neutropenia	2 (6.7)	-
10.	Peripheral neuropathy	-	4 (10.3)

In the panitumumab group, the most common adverse events were anorexia (10%), generalized weakness (10%), constipation (6.7%), and abdominal pain (3.3%). In the bevacizumab group, generalized weakness (23.1%), constipation (15.4%), anorexia (10.3%), and abdominal pain (5.1%) were the most frequently reported events. Overall, adverse events were more numerous and occurred more frequently in the bevacizumab group than in the panitumumab group.

## Discussion

CRC is a major public health concern and is linked to high rates of morbidity and mortality, along with the requirement for extensive medical treatment [21]. Over the past two decades, advances in targeted therapy and immunotherapy have enabled patients with mCRC to experience longer periods without disease progression. However, despite the development of highly effective treatment alternatives, most patients continue to struggle with incurable mCRC [22]. We conducted this study to investigate the potential benefits of targeted therapy for mCRC patients in India, as there is a dearth of information on the real-world use of targeted therapies in this country. This study is unique in that, beyond evaluating the safety and efficacy of bevacizumab and panitumumab, it also examines treatment response in relation to tumor sidedness.

The demographics of our study cohort, including mean age, gender distribution, and tumor sidedness, align with findings from earlier studies [3,23-26]. Previous reports have shown that patients under 40 years of age have significantly lower median OS [23], and that the liver is not only the most common but often the earliest site of metastasis in CRC [23,25]. Additionally, liver metastasis has been shown to significantly impact survival outcomes in mCRC patients [27]. The most frequent site of metastasis in our study cohort was the liver (66.70%), followed by the lungs, lymph nodes, peritoneum, bones, and other locations. In this study, patients with mCRC had elevated carcinoembryonic antigen (CEA) levels.

A previous study reported an inverse correlation between the median OS and the CEA level at diagnosis in individuals with mCRC [23]. Additionally, research indicates that, despite the availability of new markers and mutation analysis, CEA remains the most reliable indicator for predicting OS [28]. After their initial diagnosis, 30.4% of the patients in this study underwent surgical resection. According to a previous study, operability affects OS; therefore, patients with CRC who received surgical resection, either at diagnosis or during neoadjuvant treatment, had a longer median OS [23]. The assessment of RAS (KRAS and NRAS) and BRAF alterations is the preferred method for mCRC molecular characterization to determine the optimal treatment plan. Regulatory bodies such as the US FDA and the European Medicines Agency also advise hotspot mutation screening for KRAS, NRAS, and BRAF, before choosing a treatment approach for mCRC patients [29].

In this study, 89.8% of patients were classified as KRAS WT, and there were no patients with NRAS or BRAF mutations. Previous research has shown that NRAS mutations are uncommon (5-10%), but KRAS mutations are seen in over 40% of mCRC [30-31]. Additionally, it is well known that left- and right-sided CRCs have distinct profiling features, such as mutational profiles, genomic patterns, histology, carcinogenesis, and molecular pathways, because of genetic and epigenetic variations [32]. In our study, we discovered that patients who had tumors on the left side had a higher rate of KRAS WT (67.9% vs. 32.1%), NRAS WT (70.7% vs. 29.3%), and BRAF WT (73.2% vs. 26.8%) molecular subtypes than those who had tumors on the right side. Also, both HER2-positive individuals in our study had left-sided tumors. Studies have indicated that CRCs on the left have higher expression of RAS WT and amplification of EGFR and ERBB2 (previously HER2), while those on the right are more likely to have increasing MSI and a greater frequency of KRAS and BRAF mutations [32-33].

In the bevacizumab-based regimen, 69.23% (PR) and 87.18% (PR: 69.23%, SD: 17.95%) of the participants in this study achieved ORR and CBR, respectively. Previous studies confirm these conclusions, with 67.3% of those in the bevacizumab-based treatment group experiencing CR or PR [20], and 87.93% experiencing CBR [25]. Additionally, an odds ratio (OR) of 1.30 (95% CI: 1.11-1.52, p=0.001) for ORR and an OR of 1.36 (95% CI: 1.04-1.78, p=0.024) for disease control rate were found in a meta-analysis comparing bevacizumab therapy with placebo or other treatments, indicating that bevacizumab-based regimens performed better [34]. Furthermore, in the study cohort that was treated with bevacizumab, the PFS was 8.17 months, and the OS was 26.13 months. The PFS of this study is somewhat comparable to results from clinical trials (9.3-10.6 months) [14,35-36] and observational studies (8.4-10.8 months) [37-40].

Additionally, the median OS for first-line treatment was likewise consistent with those of other observational studies, such as BRiTE (22.9 months), BEAT (22.7 months), ARIES (23.5-26.3 months), FIRE-3 (22.7-28.6), and other studies (24 months) [25,38,39,41-42]. Additionally, the OS seen in this study was noticeably longer than that of the ACORN study (17.8 months) [43]. In the panitumumab-based regimen, 70% (PR) and 83.33% (PR: 70%, SD: 13.33%) of the participants in this study obtained ORR and CBR, respectively. The ORR in our study is relatively similar to that of another study that indicated that 74.9% of the overall population had CR or PR in the group receiving panitumumab-based regimens [20]. Additionally, a previous study found that 67% of patients had clinically meaningful CBR with panitumumab [44]. In our study, the median PFS for the cohort treated with the panitumumab-based regimen was 8.43 months, while the median OS could not be reached at a follow-up of 15.02 months. This study’s PFS is similar to a previous study that found a median PFS of 8.9 months for first-line therapy [23]. Furthermore, the panitumumab-mFOLFOX6 combination's PFS and OS in the KRAS WT and all RAS WT groups were 10.9 months (9.4-13) and 34.2 months (26.6-NR), as well as 13 months (10.9-15.1) and 41.3 months (28.8-41.3), respectively, according to the PEAK trial [42].

Compared to anti-VEGF therapy, the study's findings showed an OS advantage for first-line anti-EGFR therapy, particularly on extended RAS analysis [42]. The ORR (p=0.94), CBR (p=0.65), and PFS (p=0.1478) of the overall population in the present study did not significantly differ between the bevacizumab and panitumumab-based regimens. A meta-analysis supported the findings of this study, showing that bevacizumab and panitumumab therapies seemed to have comparable PFS (hazard ratio (HR): 0.96; 95% CI: 0.76 to 1.15) and ORR (relative ratio (RR): 2.06; 95% CI: 0.86 to 4.90) in the RAS WT population [45]. Additionally, the studies have revealed no significant difference in PFS between bevacizumab and panitumumab (PFS: 12.2 vs. 11.4 months; HR: 1.05; 95% CI: 0.90-1.24) in the general populations [20,46].

In our study, a higher percentage of patients treated with panitumumab had tumors on the left side, whereas a slightly higher number that were treated with bevacizumab had tumors on the right side. The remarkable improvements in tumor response, PFS, and OS shown with cetuximab and panitumumab have raised the possibility of preserving EGFR antibodies for left-sided RAS/BRAF WT tumors [32,47]. Additionally, the use of bevacizumab to treat tumors on the right side has been established by the CRYSTAL and PRIME studies [48-49]. There was no significant difference in the ORR (78.9% vs. 67.9%, p=0.40) and CBR (94.7% vs. 82.1%, p=0.20) for left-sided tumors between the bevacizumab and panitumumab groups in our study. A previous study found that 80.2% of participants in the panitumumab group with tumors on the left side had a CR or PR, compared to 68.6% in the bevacizumab group (point estimate of difference: 11.2%) [20]. However, in this study, the ORR and CBR of the bevacizumab group were slightly higher than the panitumumab group for left-sided tumors.

According to a previous trial, bevacizumab plus systemic chemotherapy decreased mortality in mCRC patients for both the left- and right-sided tumors [33]. This finding suggests that bevacizumab might be useful in treating both types of tumors. Furthermore, among patients with liver metastases and upfront unresectable left-sided RAS/BRAF WT CRC, the evidence indicates that bevacizumab is equally efficacious as anti-EGFR monoclonal antibodies. The study participants with tumors on the left side did not experience any noticeable difference in PFS (8.43 vs. 8.17 months, p=0.1383) between the two regimens. These results are consistent with other studies that reported no substantial difference in PFS (13.1 vs. 11.9 months) between panitumumab and bevacizumab for left-sided tumors (HR: 1.00; 95% CI: 0.83-1.20) [20]. Additionally, the findings of the CAIRO5, FIRE-3, and CALGB/SWOG 80405 studies also showed that in patients with tumors on the left side, the PFS did not differ significantly between anti-EGFR and anti-VEGF therapy [50-52].

On the other hand, we were unable to assess the OS differences between bevacizumab and panitumumab-based regimens for left-sided tumors, as the data did not mature to calculate the median OS in the panitumumab group. Previous work has shown that panitumumab has a much higher OS in mCRC patients with tumors on the left side than bevacizumab (HR: 0.82; 95.798% CI: 0.68-0.99, p=0.031) [53]. Moreover, studies have shown that anti-EGFRs are the recommended first-line targeted treatment for left-sided RAS/BRAF WT CRC, as evidenced by the numerically longer PFS, greater ORR, and statistically significantly longer OS [54].

Furthermore, ORR (p=0.51) and CBR (p=1.0) were not significantly different between bevacizumab and panitumumab regimens for right-sided tumors. Moreover, the panitumumab group did not achieve the median PFS or OS after a follow-up of 5.83 and 11.25 months, respectively. According to a previous study, the PFS for bevacizumab and panitumumab was 9.4 months vs. 7.2 months (HR: 1.43; 95% CI: 1.03-1.97) in patients having tumors on the right side [20]. The subgroup analysis revealed that panitumumab and bevacizumab did not significantly affect the OS (20.2 months vs. 23.2 months, HR: 1.09) of patients with tumors on the right side [20]. Anti-VEGF and anti-EGFR therapies have different toxicity profiles, according to prior studies [25]. The safety profiles of panitumumab and bevacizumab in the current study were as per the expectations. While anorexia, generalized weakness, and constipation were the most frequent adverse events, the bevacizumab group experienced more adverse events than the panitumumab group.

Limitations

This study has several limitations. Being a retrospective, non-randomized analysis, it is inherently prone to selection bias and confounding, as treatment allocation may have been influenced by physician preference, patient characteristics, and institutional practice. The small sample size (69 patients) limits statistical power, particularly for subgroup analyses such as tumor sidedness, and may explain the absence of significant differences in ORR, CBR, and PFS. The median OS was not reached for the panitumumab group due to shorter follow-up, restricting comparison of long-term outcomes. Additionally, chemotherapy regimens (FOLFOX, FOLFIRI, CAPOX) were not standardized, potentially confounding treatment effects. Molecular profiling was incomplete, with missing data for NRAS, BRAF, HER2, MSI, and MMR, and other relevant mutations, including TP53 and BRCA, were not assessed.

The study was conducted at three centers in India, limiting generalizability. Adverse events were reported without severity grading or details on dose modifications, and the statement that bevacizumab led to “more adverse events” lacks statistical comparison. Follow-up duration varied across groups, influencing the reliability of PFS and OS estimates. The analysis did not adjust for key confounders such as age, ECOG status, or metastatic burden. Finally, although tumor sidedness was evaluated, the findings could not be fully contextualized with existing evidence that EGFR inhibitors benefit left-sided RAS-WT tumors, owing to limited data, representing an important interpretative limitation.

## Conclusions

In addition to directly comparing the therapeutic efficacy of panitumumab- and bevacizumab-based regimens in Indian patients with mCRC, this study provides meaningful insights into the impact of tumor sidedness on treatment patterns and outcomes. The analysis indicated that panitumumab was more frequently used in patients with left-sided tumors harboring wild-type KRAS, NRAS, and BRAF, whereas bevacizumab was more commonly administered to patients with similarly profiled right-sided tumors. Despite these differences in treatment distribution, the study demonstrated that both biologic agents, when combined with first-line chemotherapy, resulted in comparable clinical outcomes in terms of response and disease control. These real-world findings underscore the clinical flexibility of using either agent in appropriately selected patients and highlight the relevance of tumor biology and location in guiding treatment decisions. Ultimately, the study contributes valuable practical evidence to inform the choice of biologic therapy in routine care for Indian mCRC patients, particularly in settings where individualized treatment selection is crucial.
